# Significance of microRNAs in Androgen Signaling and Prostate Cancer Progression

**DOI:** 10.3390/cancers9080102

**Published:** 2017-08-07

**Authors:** Ken-ichi Takayama, Aya Misawa, Satoshi Inoue

**Affiliations:** 1 Department of Functional Biogerontology, Tokyo Metropolitan Institute of Gerontology, 35-2 Sakae-cho, Itabashi-ku, Tokyo 173-0015, Japan; knt56ta@hotmail.com; 2 Department of Anti-Aging Medicine, Graduate School of Medicine, The University of Tokyo, Bunkyo-ku, Tokyo 113-8655, Japan; ayamsw@gmail.com; 3 Division of Gene Regulation and Signal Transduction, Research Center for Genomic Medicine, Saitama Medical University, Hidaka, Saitama 350-1241, Japan

**Keywords:** androgen, androgen receptor, miRNA, prostate cancer, epigenome

## Abstract

The androgen receptor (AR) plays important roles in prostate cancer development and prostate tumor growth. After binding to androgens, AR functions as a nuclear receptor and translocates to the nucleus to bind to specific AR-binding sites (ARBSs). AR regulates epigenetic factor recruitments to activate its downstream signaling. Although androgen deprivation therapy (ADT) is initially useful for prostate cancer patients, most patients eventually show resistance with hormone-refractory prostate cancers (HRPCs) or castration-resistant prostate cancers (CRPCs). Thus, new therapeutic strategies targeting HRPCs/CRPCs should be very important for clinical medicine as well as prostate cancer biology. Past studies have shown that mechanisms such as AR overexpression, hypersensitivity, variants and reprograming are responsible for developing HRPCs/CRPCs. These findings suggest that AR target genes will be major key factors. In this review article, we focus mainly on the androgen-regulated microRNAs (miRNAs) to summarize the contribution of miRNA-mediated pathways for prostate cancer progression.

## 1. Introduction

Non-coding RNAs (ncRNAs) are RNA transcripts that do not encode for proteins and can be divided into two major groups: small ncRNAs, between 18 and 200 nucleotides (nt) in length, and long non-coding RNAs (lncRNAs) which are larger than 200 nt. Among small ncRNAs, microRNAs (miRNAs) are evolutionally conserved single-stranded small non-protein coding transcripts of approximately 18–22 nt that post-transcriptionally regulate gene expression [1]. Generally, miRNAs bind to the 3’ untranslated region (UTR) of mRNAs to inhibit their translation. Aberrant expressions of miRNAs have been well documented in most types of cancer [2,3]. Several studies have shown the importance of miRNAs as modulators of key cellular processes in normal physiology [4,5] as well as in diseases such as cancer, including prostate cancer [6,7,8]. It is expected that some of these miRNAs could serve as future biomarkers for diagnosing cancer [9].

Prostate cancer is one of the leading causes of cancer morbidity and mortality in developed countries. Androgen receptor (AR) and its downstream signaling are fundamental for the development and progression of both localized and advanced metastatic prostate cancer [10,11]. Advanced or metastatic prostate cancer is treated with androgen deprivation therapies [12,13,14,15]. Blocking AR activity by castration or using antagonists of AR elicits a favorable response. However, most of these cancers eventually relapse and progress to hormone-refractory prostate cancer (HRPC) or castration-resistant prostate cancer (CRPC). Enhanced AR downstream signaling caused by aberrant activation of AR variants such as AR-V7, hypersensitivity to androgens, overexpression of AR and intratumoral steroidogenesis [16,17,18,19] are assumed to drive the tumor for progressing into this lethal state. Concerning variants, AR mRNA is alternatively spliced to AR-Vs and results in prematurely termination of the full AR protein. Most AR-Vs are missing the ligand binding domain (LBD), however, retain the N-terminal domain (NTD) to drive transcription androgen-independently. Among these variants, AR-V7 is expressed in HRPCs/CRPCs most frequently and could be the therapeutic target of tumors resistant to existing therapies directed to androgen/AR [20].

Thus, it is critical to investigate AR downstream-signaling or regulatory mechanisms by AR to understand how HRPCs/CRPCs develop among the patients. Therefore, identification of AR downstream signals and new molecular mechanisms for AR activation are important to improve the treatment of advanced prostate cancer [21]. In the present review, we summarize the roles of miRNAs in prostate cancer progression and CRPC development. In particular, we would like to focus on miRNAs regulated by AR or those regulating AR activity, because enhanced AR activity is important for the progression of prostate cancer.

## 2. Expressions of miRNAs in Prostate Cancer Tissue

Although several studies have investigated alteration of miRNA expression profiles in prostate cancer tissues as potential diagnostic and prognostic tools, unique signatures are still missing. In 2006, genome-wide microarray analysis performed by Volinia et al. revealed a general upregulation of miRNAs in various cancer tissues [22]. In contrast, comprehensive miRNA regulation in prostate cancer tissues compared with normal prostate is still controversial. Porkka et al. observed a general downregulation of miRNA expression in prostate cancer by custom miRNA array [23]. Various researchers also demonstrated a general downregulation of miRNAs in prostate cancer tissues [24,25,26,27,28]. Downregulation of miR-205 [27,28], miR-145 [24,27], let7 family [24,26] and miR-221/222 [27,28] were reproduced in more than two studies. Whereas, a global upregulation of miRNA has been reported in a recent analysis of high and low-grade prostate cancer and BPH by high-throughput genome sequencing [29]. In this study, upregulation of miR-125b in high-grade prostate cancer tissues were reported in line with another report [30].

miRNAs are attractive molecules as non-invasive biomarker candidates because they can be reproducibly extracted from various clinical samples. It was demonstrated that miRNAs originating in human xenografts enter the circulation and can be detected in mouse plasma [31]. miR-141 was significantly higher in patients with advanced prostate cancer than in matched controls. Differential expression analysis of 669 miRNAs in 84 serum samples from prostate cancer patients showed that expression of miR-141, miR-375 and miR-378 increased with disease progression [32]. miR-141 and miR-375 were found to be miRNAs highly expressed in metastatic CRPC patients in an analysis of 365 miRNAs in serum comparing CRPC with age-matched healthy controls [33]. It was reported that plasma miR-141 level was also shown to be associated with poor outcome, higher Gleason score and high probability of recurrence after radical prostatectomy, and that miR-21 was found to be upregulated in plasma of metastatic CRPC patients [34]. In another study, miR-21 in patients’ plasma was also found as a miRNA predicting aggressiveness of prostate cancer [35]. miR-221-5p and miR-708-3p were confirmed to be downregulated in blood of prostate cancer patients compared with benign prostate hyperplasia (BPH) patients by quantitative real-time PCR (qRT-PCR) [36]. Ambs et al. reported that miR-106b and miR-32 were upregulated in prostate cancer tissues [37]. miR-106b cluster expression is associated with early disease recurrence and targets *caspase-7*, *E2F1* or *p21* [38]. Both miR-106b and miR-32 are androgen-regulated [39,40]. Taken together, these reproduced results indicate the importance of miR-141, miR-375, miR-32, miR-21 and miR-106b in CRPC development.

It is still controversial whether miR-221/222 expression promotes CRPC. Several groups have reported that miR-221/222 was highly expressed in bone metastatic CRPC tumor samples [41] and that high expression of miR-221 was observed in castration-resistant cell lines [42]. Functionally, miR-221/222 has been reported to promote androgen-independent cell growth [43]. Overexpression of miR-221/222 in LNCaP cells induced development of CRPC by targeting *HECT domain E3 ubiquitin protein ligase 2 (HECTD2)* and *RAB1A* [43,44,45]. Another study demonstrated that cell cycle progression is induced by miR-221/222 by targeting *p27*, cyclin dependent kinase inhibitor [46]. In contrast, several studies reported that the expression of miR-221/222 was downregulated in prostate cancer and CRPC clinical samples [27,47,48,49]. We can speculate that the role of miR-221/222 may not be simple and that it may depend on the environment of tumor whether they function as tumor-suppressive miR or onco-miR.

Other recent reports using a combination of DNA microarray datasets identified miRNAs that may contribute to CRPC development [50]. Among them, miR-149, miR-197, miR-210, miR-218 and miR-346 were found to be overexpressed while miR-122, miR-145 and let-7 were under-expressed in CRPC cell lines compared to androgen-sensitive prostate cancer cells [50]. Pathway analyses revealed that miR-218, miR-197, miR-145, miR-122, and let-7b, along with their target genes, Ras, Rho proteins and Skp, Cullin, F-box (SCF) complex, were involved in the PI3K and AKT3 signaling network, which is known to contribute to CRPC development. Interestingly, the tumor suppressor miR-145 was found to bind prostate cancer gene expression marker 1 (PCGEM1), decreasing its expression, while downregulation of *PCGEM1* increased miR-145 expression [51].

## 3. Cancer Stem Cells Regulated by miRNAs

Cancer stem cells (CSCs) are an important subset of cancer cells that contribute to tumor progression and metastasis in various cancers. Prostate CSCs could be found by using the adhesion molecule CD44 as a marker, as CD44 was shown to increase potential for tumor progression [52]. Several groups found that miRNAs have key roles in regulating CD44 expression. miR-34 has been shown to be repressed in CD44+ prostate cancer cells [52]. By inhibiting miR-34, cancer cells promoted tumor development and metastasis [52]. CD44 is a direct and functional target of miR-34a, suggesting the key regulatory role of miRNAs in the development of CSCs in prostate cancer [53]. In addition to miR-34, miR-141 was identified to suppress prostate CSCs by targeting a cohort of pro-metastasis genes such as *CD44*, *Enhancer of Zeste Homolog 2 (EZH2)* and Rho GTPase family members [54]. Interestingly, the authors found that the expression of miR-141 is downregulated in prostate CSC populations of primary patients’ tumors, although miR-141 is highly expressed in prostate cancer tissues [54]. These findings indicate that regulation of CSCs by miRNAs would be a useful strategy to obstruct tumor growth and metastasis.

## 4. Identification of Androgen-Regulated miRNAs in Prostate Cancer

Several studies have identified androgen-regulated miRNAs, which may act as oncogenic miRNAs or tumor suppressive miRNAs. Among them, in one of the early studies reporting androgen-regulated miRNAs, miR-125b abundance was measured in clinical prostate cancer samples by in situ hybridization (ISH) [30]. Compared with the benign prostatic tissues, five prostate cancer samples highly expressed miR-125b, and four exhibited a moderate increase in miR-125b [30]. Androgen treatment up-regulate the expression of miR-125b in prostate cancer cell lines [30]. Transfection of synthetic miR-125b stimulated androgen-independent growth of prostate cancer cells and down-regulated the expression of *B-cell lymphoma 2 antagonist/killer 1 (Bak1)*, which is known to induce apoptosis, suggesting that miR-125b acts as an oncogenic miRNA [30].

A microarray analysis on AR-positive prostate cancer cell lines identified 16 AR-responsive miRNAs [55]. qRT-PCR analysis revealed elevated miR-21 expression in prostate cancer tissues compared with adjacent benign tissues [55]. After androgen stimulation, AR is recruited to miR-21 promoter region, suggesting direct transcriptional regulation by AR. Inhibition of miR-21 diminished androgen-induced cell proliferation, while overexpression of miR-21 enhanced tumor growth in vivo [55]. Moreover, this overexpression was sufficient for androgen-dependent tumors to overcome castration-mediated growth arrest, suggesting that elevated miR-21 expression alone is sufficient to impart castration resistance [55]. *Transforming growth factor, beta receptor 2* (*TGFBR2)*, *Programmed cell death protein 4 (PDCD4), Reversion-inducing cysteine-rich protein with kazal motifs (RECK), p57kip2* and *Phosphatase and Tensin Homolog Deleted from Chromosome 10* (*PTEN)* have been reported as miR-21 targets in prostate cancer [56,57,58,59,60].

**Figure 1:**
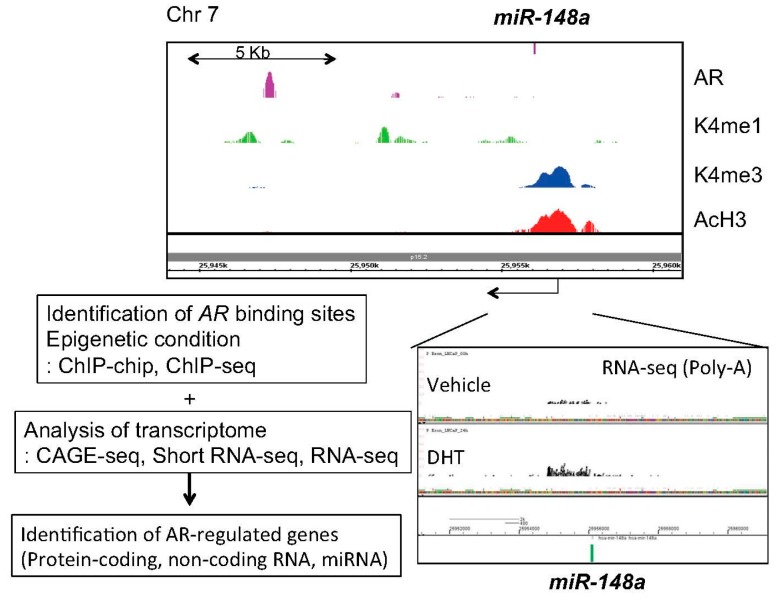
Investigation of AR-mediated miRNA regulation by integrating epigenetic and transcriptomic approaches. We performed CAGE, RNA-seq, short RNA-seq and ChIP-seq to reveal comprehensive epigenetic status, androgen-regulated transcripts and AR binding sites. CAGE; Cap analysis of gene expression, K4me1 or 3; Histone H3 lysine (K) 4 mono or tri-methylation, AcH3; Acetylated histone H3.

Next, in our short RNA sequencing (RNA-seq) studies, 11 androgen-regulated miRNAs, including miR-148a, miR-106b and miR-29a/b, which are common with the previous microarray study [55], were identified [39]. Among them, miR-141, miR-200a and miR-148a were validated as androgen-induced miRNAs in LNCaP cells [39]. By using knockdown and overexpression analyses, we demonstrated that these miRNAs promote prostate cancer cell proliferation [39]. Mechanistically, we showed that miR-148a reduced the expression of *cullin-associated and neddylation-dissociated 1 (CAND1)* by binding to the 3'-UTR region of the mRNA [39]. *CAND1* knockdown by siRNA promoted cell proliferation suggesting the potential contribution of miR-148a to the growth of human prostate cancer [39]. We have integrated several genomic approaches to investigate AR downstream signaling using next generation sequencing [6,21,61]. Further analysis using these data [6,21,61] found histone modification of miR-148a promoter and AR bindings in the vicinity, suggesting that this miRNA is a direct AR target (Figure 1). Clinical studies have revealed that high miR-148a expression is significantly correlated with prostate cancer progression [40,62,63]. Consistent with our finding, subsequent microarray analyses in prostate cancer cell lines and xenograft models have revealed that miR-141 is overexpressed in prostate cancers and CRPCs compared to BPH tissues [64]. Overexpression of miR-141 enhanced growth of parental LNCaP cells while inhibition of miR-141 suppressed growth of the LNCaP subline overexpressing AR [39,64]. Moreover, miR-27a was also found to be androgen-regulated in our study [39]. Another group validated this regulation and showed that miR-27a promotes prostate cancer cell proliferation by targeting *ATP-binding cassette transporter 1* (*ABCA1)* and *PDS5 cohesin associated factor B* (*PDS5B)*, suggesting the oncogenic role of miR-27a [65]. In this report, other two miRNAs, miR-19a and miR-133b, were newly recognized as novel AR targets [65]. Overexpression of miR-133b promoted prostate cancer cell proliferation [65]. Furthermore, miR-133b and its target, *RB1-inducible coiled-coil protein 1 (RB1CC1)*, were reported to be independent prognostic factors for prostate cancer patients [66].

The investigation of miRNA expression profiling in clinical CRPC samples found that seven miRNAs were differentially expressed in CRPCs by microarray analyses [40]. Among them, miR-32, miR-148a and miR-21 were found to be significantly overexpressed, and miR-99a, miR-99b and miR-221, significantly underexpressed in CRPCs. Western blotting and 3′-UTR-luciferase assays using LNCaP cells transfected with pre-miR-32 and pre-miR-148a revealed that *BTG Anti-Proliferation Factor 2 (BTG2)* and *phosphoinositide-3-kinase interacting protein 1 (PIK3IP1)* are targets of these miRNAs, respectively. Furthermore, their immunohistochemical analysis revealed a significant reduction of BTG2 protein in CRPCs compared with untreated prostate cancer. Additionally, lack of BTG2 staining was associated with a short progression-free time in patients who underwent prostatectomy, which makes miR-32 a potential biomarker of CRPC [40]. Recently, miR-193a-3p was also shown to be androgen-induced and overexpression of miR-193a-3p promotes CRPC cell proliferation by targeting Ajuba LIM Protein (AJUBA), which is repressed in metastatic prostate cancer tissues [67].

Furthermore, miR-200a and miR-200b were identified as androgen-regulated miRNAs [39,68]. We analyzed the effect of miR-200a with knockdown and addition of miR-200a in LNCaP cells and showed a positive role of miR-200a in proliferation of AR-positive prostate cancer cells [39]. In contrast, it was reported that overexpression of miR-200b inhibits prostate cancer proliferation and tumor growth by reversal of the epithelial-to-mesenchymal transition (EMT) [68]. Additionally miR-200c was shown to be repressed by TMPRSS2-ERG fusion proteins and downregulated in ERG-positive prostate cancer [69]. ERG-mediated repression of miR-200c activates *Zinc Finger E-Box Binding Homeobox 1 (ZEB1)* expression, which is responsible for EMT. Another study demonstrated that miR-200 family targets *snail family transcriptional repressor 2 (SNAI2/SLUG)* directly to inhibit EMT and functions as tumor suppressive miRNAs [70]. Nevertheless, all of these miR-200 family members could be detected in serum of CRPC patients and high expression of miR-200 family is proposed to be a prognostic factor of CRPC [71,72]. Although there are controversies among miR-200 functions, these findings suggest the importance of miR-200 family in EMT, cell proliferation and disease progression of prostate cancer.

Moreover, tumor suppressive roles of androgen-regulated miRNAs have also been reported [73,74,75]. We first identified miR-99a as androgen-regulated miRNA by short RNA sequence and qPCR analysis [39,61]. Several studies reported that reduction of miR-99a in CRPC compared to benign prostate tissues [40,75]. Overexpression of miR-99a suppresses prostate cancer cell proliferation. In addition, miR-135a was identified as an androgen-regulated miRNA by highthroughput RT-PCR analysis [73]. miR-135a overexpression decreased in vivo invasion abilities of PC-3 cells. This effect is mediated by directly targeting *Rho Associated Coiled-Coil Containing Protein Kinase 1 (ROCK1)* and *ROCK2* expression [73] or AR activity [76]. It was reported that miR-30 family members are also androgen-induced [39]. Both miR-30-b and miR-30d negatively regulate AR signaling by directly targeting 3′-UTR of *AR* mRNA [74]. Expressions of both miRNAs are reduced in metastatic CRPC tissues [74]. Thus, androgen-regulated miRNAs have important roles in controlling tumor growth and EMT by modulating cancer-related pathways.

## 5. Regulation of Androgen Signaling by miRNAs

**Figure 2:**
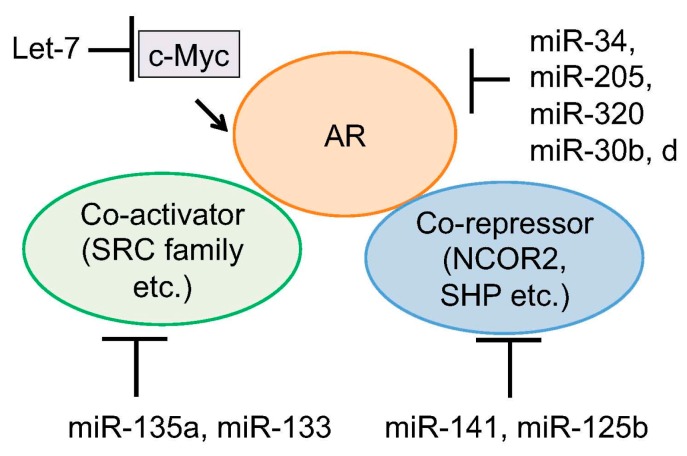
Regulation of AR activity by miRNAs. Several miRNAs were reported to directly target AR mRNA to reduce AR protein level. Another mechanism is to inhibit expression level of AR coregulators or associated transcription factors by miRNAs.

The major important target of miRNAs in prostate cancer is AR signaling. Targeting AR-associated factors or AR itself by binding to the 3′-UTR of mRNAs has been reported (Figure 2). Coarfa et al. reported that expression of 12 miRNAs (miR-1, miR-133a, miR-133b, miR-135a, miR-143-3p, miR-145-3p, miR-205, miR-221-3p, miR-221-5p, miR-222-3p, miR-24-1-5p and miR-31) was suppressed in metastatic prostate cancer [76]. They found that AR and members of steroid receptor coactivator family (SRC family) are major targets of these miRNAs. In addition, they also found that miR-135a is robustly induced by androgen and that strong direct binding of AR was observed around the miR-135a locus. This regulation is involved in miR-135a-mediated suppression of AR and AR expression could be restored under androgen-depleted condition, contributing to the upregulation of AR in CRPC [76].

Members of miR-34 family (-a, -b, -c) were reported to suppress tumorigenesis by several mechanisms including regulation of cell cycle, EMT and/or metastasis [77,78,79,80,81]. In cancer, p53, a major tumor suppressive transcription factor, induce miR-34 family [82]. In prostate cancer, miR-34 family members are downregulated and miR-34a or miR-34c correlated with the tumor grade, advanced disease, and life expectancy of patients. In addition, miR-34a expression level is inversely associated with AR, suggesting the repressive role of miR-34 family in AR expression [83]. By using protein lysate microarrays, systematic analysis of miRNAs targeting AR was performed. In this report, 3′-UTR-binding assays revealed 13 miRNAs (such as miR-34a and miR-34c) that bind to and regulate 3′-UTR of *AR* [83].

Let-7 levels are known to be downregulated in cancers [24]. Let-7c targets the oncogenes such as *Ras* and *Myc* and suppresses prostate cancer progression through AR by targeting *c-Myc*, which is required for AR function [84]. Moreover, let-7c reduces AR activity and inhibits CRPC cell growth by associating with *c-Myc* 3′-UTR and subsequent reduction of *AR* transcription [84].

miR-205 is deregulated in prostate cancer tissues compared with benign prostate tissues. It is inversely associated with advanced disease and poor outcome of prostate cancer patients, and miR-205 levels in cancer tissues exhibit a negative correlation with AR immunoreactivity [76,85]. In addition, miR-205 levels in cancer tissues was found to be lower in CRPC patients compared with those in men who had not treated with hormone therapy [85,86]. Moreover, it has been found that miR-205 regulates target genes such as *IL-8* related with AR or B-cell lymphoma 2 (BCL2), MAPK/ERK, mTOR and IL-6 signaling genes. Additionally, other miRNAs such as miR-30b, miR-30d [74], miR-31, miR-124a, miR-320 [87] and miR-212 [88] were also identified to directly inhibit AR expression. Interestingly, miR-320 mediates the effect of histone deacetylase inhibitor in prostate cancer by targeting AR expression [87]. Thus, we can expect that inhibition of these miRNAs directly targeting *AR* expression is an important pathway for development and progression of prostate cancer.

In addition to the tumor suppressive miRNAs, oncogenic role of miRNAs to activate AR signaling has been documented. As described above, expression of androgen-induced miR-141 is associated with the tumorigenesis of prostate cancer [32,33]. The *orphan receptor small heterodimer partner (SHP)* is a co-repressor of AR and represses AR-activity [89]. miR-141 associated with 3′-UTR of *SHP* and suppresses the mRNA expression. Thus, miR-141 promotes AR transcriptional activity [89]. Moreover, another AR corepressor, nuclear receptor corepressor 2 (*NCOR2)*, is a target gene of miR-125b, which is induced by androgen [90]. *NCOR2* repression enhances AR transcriptional activity [90].

## 6. Targeting Epigenetic Condition by Androgen-Regulated miRNA

**Figure 3:**
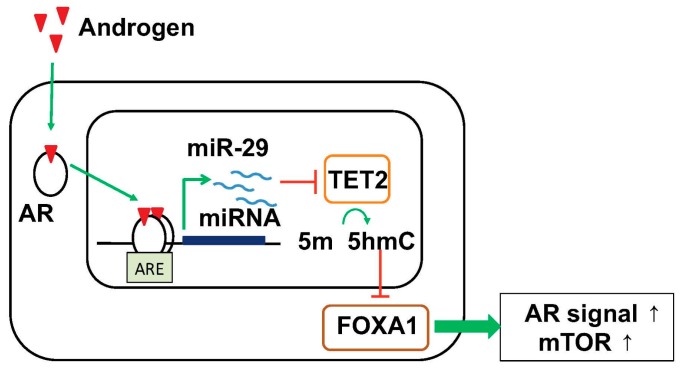
Model of TET2 repression by androgen-induced miR-29 to control the genome-wide 5-hydroxymethylated cytosine (5-hmC) status for prostate cancer progression. miR-29 represses TET2, causing reduction of 5-hmC and subsequent reduction of FOXA1 binding to specific binding sites, consequently activating pathways related to prostate cancer progression such as AR signaling and mTOR pathways. AR, androgen receptor; 5-hmC, 5-hydroxymethylated cytosine; 5-mC, 5-methylated cytosine; ARE, androgen responsive element.

We analyzed the function of AR-responsive miRNAs in prostate cancer cells and proposed a novel working model that androgen-induced miRNAs functions as “epigenetic modifiers” and regulate the global epigenetic condition for progression to HRPCs/CRPCs (Figure 3) [91]. Our short RNA-seq analysis in HRPC cell models found several androgen-induced miRNAs. Among them, two miRNAs, miR-22 and miR-29 family, are robustly induced by androgen treatment in this cell model [91]. Our analysis of miR-29a/b expression by in situ hybridization (ISH) in clinical prostate cancer tissues revealed that they are expressed higher in tissues associated with poor prognosis and high Gleason scores [91]. Importantly, miR-29a/b target *ten–eleven translocation 2 (TET2)*, an enzyme that catalyzes the oxidation of methylated cytosine (5-mC) to 5-hydroxymethylated cytosine (5-hmC), an epigenetic hallmark of prostate cancer progression [91]. In clinical samples, immunohistochemical analysis revealed that downregulation of 5-hmC and TET2 was found to be associated with poor outcome of the patients [91]. Moreover, 5-hmC modifications by TET2 decreased the bindings of Forkhead box protein A1 (FOXA1) and subsequent AR enhancer activity [91] since FOXA1 is a pioneer factor inducing AR recruitments [92]. Meanwhile, reduction in 5-hmC by miR-29a/b activated prostate cancer-related key pathways such as mammalian target of rapamycin (mTOR) and AR, indicating significant oncogenic roles of miR-29 in prostate cancer progression [91]. Notably, another study also observed the importance of TET2 in prostate cancer by regulating AR activity [93]. Moreover, recent genome-wide association studies in prostate cancer tissues have demonstrated that *TET2* variation is genetically correlated with the development of prostate caner and metastasis [94,95]. Importantly, in other studies in breast cancer and myelodysplastic syndrome (MDS), it was suggested that miRNA-mediated TET2 repression contributes to tumorigenesis [96,97]. It was also reported that miR-22 and miR-29 function as onco-miRs by regulating epigenetic status in cancer [98,99]. In addition to these two miRNAs, several androgen-regulated miRNAs such as miR-125b and miR-200a repressed *TET2*-3′UTR, suggesting *TET2* is a common target of these AR-induced miRNAs [91]. In line with these miRNA inductions with androgen, TET2 expression is repressed by androgen treatment [91]. Thus, TET2 regulation by androgen-induced miRNAs would be a key event for modifying epigenetic condition in cancer cells. We speculate that these epigenetic changes would be one of triggers for the development of HRPCs.

We examined the expression of miR-29a and miR-29b in prostate cancer tissues. Our analysis revealed higher expression of these miRNAs in prostate cancer tissues is associated with poor prognosis of prostate cancer patients. In contrast, analysis of the miRNA signature of prostate cancer compared with benign tissues showed that expression of miR-29 family members including miR-29b is significantly downregulated in cancer tissues compared to normal prostate tissues [100]. Although the role of miR-29 family in cancer progression is still controversial, miR-29a promotes breast cancer metastasis [101], and miR-29b enhances NF-kB pathway in lung cancer [102]. miR-29b is dysregulated in lung cancer for modulating epigenetic status [99,102]. These findings indicate the roles of miR-29 family should be determined dependent on the environment or tumor type. The miRNAs could present different functional importance as both tumor suppressive or oncogenic miRNAs in clinical course.

## 7. Clinical Application of miRNA for Prostate Cancer Diagnosis and Therapy

The high stability of miRNAs [31] and abnormal circulating miRNA profiles in cancer patients [103] have raised the possibility that they could be used as useful prognostic biomarkers in various cancers including prostate cancer [104]. Several studies have investigated the potential of circulating miRNAs as therapeutic response biomarkers in metastatic prostate cancer [32,33,34,105,106]. These studies demonstrated that miR-141, miR-200a/c, miR-210, miR-375 and miR-21 are oncogenic miRNAs used for biomarkers in serum from metastatic CRPC patients. However, these studies were limited by the size of patient cohorts and the number of miRNAs tested.

Lin et al. investigated biomarkers by analyzing circulating miRNA in 97 CRPC samples. High levels of miR-21, miR-200 family (miR-200a, miR-200b, miR-200c, miR-375) and low levels of miR-17 family (miR-20a, miR-20b) or miR-222 were associated with PSA-response and shorter survival of patients. High levels of miR-200 family members are associated with poor chemotherapy outcome of patients in this study [71]. This group recently reported the result of phase II study and validated the importance of miR-200 family in 89 CRPC patients [72]. A possible role of miR-200 family in CRPC is enhanced metastatic ability, as observed in breast cancer [107]. miR-200s promoted metastatic colonisation of breast cancer cells through its gene target *Sec23 (S. Cerevisiae) Homolog A (SEC23A).* In addition, we have also reported that miR-200 families are androgen-responsive as mentioned [39], suggesting that AR have a role for activating these miRNAs and promotes prostate cancer cell proliferation. 

In addition, there is an increasing interest in the application of exosomes as non-invasive biomarkers [108]. Exosomes play an important role in intercellular communication through transfer of various molecules (RNAs, DNA and proteins). They are secreted membrane vesicles that are 30–100 nm in size and actively present in nearly all body fluids [109]. In prostate cancer, a recently developed non-invasive urine test, 3-gene expression assay in exosomes, has demonstrated an improved identification of patients with higher-grade prostate cancer among men with elevated PSA reduce the number of unnecessary biopsies [110]. Analysis of the transcriptome in tumor exosomes isolated from the urine of patients with prostate cancer revealed biomarkers with potential for cancer diagnosis [78,111]. Analysis of miRNAs will help to classify the tumor phenotype, its severity and the tumor response to treatment. Alteration of certain specifics miRNAs, such as mir-107, mir-574-3p and mir-483-5p, was found in the urine of men with prostate cancer compared with healthy controls [111]. Exosomal levels of miR-34a were significantly reduced in the urine of prostate cancer relative to BPH [78]. Although only a few exosomal biomarkers have been developed, we expect that the development of accurate isolation and detection methods will trigger the application of novel exosomal biomarkers into the clinical practice in the near future.

Effective delivery of miRNAs to cancer cells is very challenging because of the lack of miRNA trapping system to cancer cells [112]. Moreover, miRNAs are rapidly degraded and excreted in serum condition. As we described above, miR-34 has an important roles in CSC development and metastasis in cancer. Liposomal miR-34 mimic (MRX34) is in under clinical study in several cancers such as lung cancer [113]. Furthermore, a recent study demonstrated that targeting miR-34 also have efficacy for treating CRPC [114]. Complete or partial genomic loss of a locus, chr13q14, in which miR-15a and miR-16-1 are located, was reported in advanced prostate cancer [115]. This genomic loss is associated with metastasis and tumor progression [116,117]. These miRNAs target several oncogenes such as *BCL2, Cycline D1 (CCND1)* and *WNT3A* in prostate cancer. Based on this mechanism, miR-16-conjugated atelocollagen has been shown to inhibit bone-metastatic human xenograft growth in mouse bone site in vivo [118]. In addition to these trials, inhibition of oncogenic miRNAs could also become another next-generation strategy for treating CRPC. 

## 8. Summary

The main priority in clinical prostate cancer research is identifying novel biomarkers to reliably distinguish between low-risk patients and high-risk patients who need definitive treatment. Strong evidences have been presented about the significance of miRNAs in prostate cancer (summarized in Table 1). The following conclusions may be put forth:

(1) AR-regulated miRNAs such as miR-21, miR-32, miR-125b, miR-141, miR-148a promoted tumor growth by regulating downstream signals such as cell cycle, apoptosis and invasion. These miRNAs are upregulated in CRPC or metastatic cancer.
(2) Several miRNAs target *AR* or *CD44* directly to inhibit development of tumor and cancer stem cells. Loss of these miRNAs may be critical step for prostate cancer progression.
(3) Changes of global epigenetic code by AR-regulated miRNA induction would be important pathway for inducing HRPC and promotes tumor growth.
(4) The functions of miRNAs such as miR-141, miR-221/222 and miR-29a/b are not unique during prostate cancer progression.

We should therefore consider the cell/tumor type or stage and expression levels of their target genes to evaluate the roles of these miRNAs. Although recent studies showed that measurement of these transcripts in clinical samples could improve the prediction of prognosis, more understanding of molecular events that lead to metastatic prostate cancer would enable us to determine treatment modalities at the time of diagnosis.

## Figures and Tables

**Table 1:** Summary of representative miRNAs reported to be important in the prostate cancer progression.

miRNA	Expression in PCa vs Benign	Functions
miR-34a	Low in CSCs	Tumor suppressive. Targets AR, CD44 and EZH2. miR-34a loss promotes development of cancer stem cells. [52,53,77,78,79,80,81,82,83]
miR15a/16	Low	Inhibits cell proliferation and invasion. Targets BCL2 and CCND1. [116,117]
miR-205	Low	Targets AR. Inhibits cell proliferation.[85]
let-7c	Low	Targeting c-Myc and subsequently inhibits AR activity. (84)
miR-135a	Low	Regulated by androgen. Targets ROCK1, ROCK2, AR and SRC family. Inhibits cell proliferation. [73,76]
miR-320	Low	Induced by HDAC inhibitor. Targets AR. [87]
miR-145a	Low	Targets PCGEM1 (51). Decreased in CRPC. [50]
miR-200a, b, c	Low	High expression in plasma is associated with poor prognosis. Inhibits EMT by targeting ZEB1, SNAIL and SLUG. Androgen-regulated and promotes cell proliferation. [39,68,69,70,71,72]
miR-221/222	Up in CRPC Low	Targeting HECTD2. Promotes CRPC cell growth. Induce cell cycle by targeting p27. [41,42,43,44,45,46]
miR-29	Up in HRPC Low	Higher expression is associated with poor prognosis. Global 5-hmC status by targeting TET2. Enhance FOXA1 and AR signals. AR-regulated miRNA. [91]
miR-125b	High	Oncogenic miRNA. Targets Bak1, NCOR2 and inhibits apoptosis. Direct AR target miRNA. [30,90]
miR-21	High	Increases with disease progression. Highly expressed in plasma of advanced PCa. Direct AR target miRNA. Targets PDCD4, RECK, p57kip2 and PTEN. [55,56,57,58,59,60]
miR-141	Low in CSCs High	AR-regulated miRNA. Associated with CSC development. Promtoes cell growth and metastasis. [34,39,64] Increase with disease progression. Activate AR activity by targeting Corepressor, SHP [89]. Inhibits metastasis and growth by targeting pro-metastasis genes [54].
miR-32	High	AR-regulated miRNA. Upregulated in CRPC. Targets BTG2. [40]
miR-148a	High	AR-regulated miRNA. Promotes cell proliferation. Targets CAND1 and PIK3IPI. [39,40]
miR-375	High	Increases with disease progression. Highly expressed in plasma of advanced PCa. [32,71]
miR-133b	High	Induced by androgen. Targets RB1CC1. Independent predictor for recurrence. [65,66]
miR-27a	High	Androgen-regulated. Targets ABCA1, PDS5B. Promotes cell proliferation. [39,65]
miR-30b, d	Low	Reduced in CRPC tissues. Targets AR. [74]
miR-99a	Low	Androgen-regulated [39,61]. Reduced in CRPC. [40,75]

PCa: prostate cancer, CSC: cancer stem cell, HDAC: histone deacetylation inhibitor.
